# Evaluating the Psychometric Properties of a Scale to Measure Perceived External and Internal Faces of Controlling Teaching among Students in Physical Education

**DOI:** 10.3390/ijerph18010298

**Published:** 2021-01-03

**Authors:** Rafael Burgueño, Ángel Abós, Luis García-González, Henri Tilga, Javier Sevil-Serrano

**Affiliations:** 1 Health Research Centre, University of Almeria, 04120 Almeria, Spain; rmburgueno@ual.es; 2 Department of Physical Education and Sport, Faculty of Sport Sciences, University of Granada, 18071 Granada, Spain; 3 EFYPAF “Physical Education and Physical Activity Promotion” Research Group, Faculty of Social and Human Sciences, University of Zaragoza, 44002 Teruel, Spain; aabosc@unizar.es; 4 EFYPAF “Physical Education and Physical Activity Promotion” Research Group, Faculty of Health and Sport Sciences, University of Zaragoza, 22001 Huesca, Spain; jsevils@unizar.es; 5 Institute of Sport Sciences and Physiotherapy, Faculty of Medicine, University of Tartu, Ujula 4 Str., 51008 Tartu, Estonia; henri.tilga@ut.ee

**Keywords:** motivating style, need-thwarting behaviours, controlling behaviour, externally controlling, internally controlling, basic psychological needs

## Abstract

There are no validated instruments to date that have examined the students’ perceptions of externally and internally controlling teaching practices in physical education (PE). Grounded in self-determination theory, the objective of this research was to provide validity and reliability evidence of the Controlling Teaching Scale for Physical Education (CTS-PE) to assess the external and internal faces of controlling teaching in PE through two sequential studies. In Study 1 (*n* = 241 students), an exploratory factor analysis revealed an eight-item two-factor solution (four items per factor). In Study 2 (*n* = 968 students), a confirmatory factor analysis supported the eight-item two-factor correlated model (i.e., externally and internally controlling teaching) that was invariant across gender. Reliability coefficients indicated an acceptable level of reliability for the two factors of the CTS-PE. A structural equation modelling showed that externally and internally controlling teaching behaviours positively predicted need frustration, and negatively need satisfaction. The current study gathered evidence to consider the CTS-PE as a valid and reliable instrument to assess students’ perceptions of PE teachers’ externally and internally controlling teaching behaviours. The CTS-PE provides PE teachers with deeper insights into the negative psychological experiences associated with externally and internally controlling teaching behaviours in PE.

## 1. Introduction

In the school physical education (PE) setting, the teaching behaviours of the teachers can play a key role in shaping students’ learning and motivational experiences involved in the teaching and learning process [1,2]. Specifically, controlling teaching behaviours have been found to be positively associated with maladaptive psychological experiences and outcomes in PE [3,4,5]. Thus, to inform PE teachers about the potential risks derived from exhibiting controlling teaching behaviours in their classes, it is necessary to provide more detailed insights into the nature and distinct manifestations of this type of control practices. Grounded in self-determination theory (SDT) [6], De Meyer et al. [7] evidenced that there may be two clearly differentiated faces (i.e., external and internal) of controlling teaching behaviours. Although De Meyer et al. [7] developed the Controlling Teaching Scale for Physical Education (CTS-PE) to assess students’ perceptions of externally and internally controlling teaching behaviours from PE teachers, its psychometric properties still remain to be examined. Therefore, the goal of this SDT-based research was to gather validity and reliability evidence of the CTS-PE as a psychometrically robust measure to differentially assess PE teachers’ externally and internally controlling teaching behaviours perceived by students.

### 1.1. Self-Determination Theory and Controlling Behaviours in Physical Education

Consistent with the SDT perspective, controlling teaching behaviours are characterised by those behaviours that focus on ignoring students’ viewpoints, pressuring them to think and behave in a specific manner, using rigorous standards as a reference, and making them feel that they must strictly comply with their teachers’ instructions [8,9] Research guided by SDT has suggested that teachers’ controlling teaching behaviours can be understood in terms of at least two distinguishable faces, depending on its nature: an externally controlling teaching and an internally controlling teaching [10,11].

Externally controlling teaching refers to classroom social environments where students feel forced to strictly fulfil requirements and demands imposed by the teachers [11]. For instance, teachers, who develop externally controlling teaching behaviours, adopt strategies focused on activating a sense of external duty and obligation in students by relying on tangible external contingencies such as the use of an explicitly controlling language (e.g., “you should” or “you must”), yelling, surveillance, rewards, incentives, and deadlines, as well as threats and punishments [7,10,12]. For example, when the PE teacher forces students to carry out squats because they behave in a disruptive manner in the lesson. In contrast, internally controlling teaching refers to classroom social environments where students feel internally pressuring forces to commit to the behaviour requested by the teacher. To illustrate, teachers, who implement an internally controlling teaching, use strategies aiming to appeal to students’ feelings of self-esteem, shame, guilt, and anxiety [7,10,12]. Indeed, internally controlling teaching strategies sometimes may occur in relatively subtler and less directly observable manners, for example, by means of the withdrawal of attention or facial displays of disappointment, while at other times they could also be exhibited in an overt and open way, for instance, through verbal expressions of disapproval [7,12].

Regardless of the nature of controlling teaching behaviours, SDT [8,13] holds the assumption that controlling behaviours may undermine the satisfaction of the basic psychological needs (BPN) for autonomy (i.e., experiences of volition and choice in the actions undertaken), competence (i.e., experiences of efficacy and mastery to accomplish expected outcomes), and relatedness (i.e., experiences of mutual care and feel part of a group). Controlling teaching behaviours, in turn, hinder the optimal development of autonomous motivation (i.e., behavioural regulations based on experiences of enjoyment, personal value, and psychological freedom), and adaptive affective, behavioural, and cognitive consequences [8,13]. Indeed, this type of teaching behaviours is postulated to lead directly to experiences of frustration of the BPN for autonomy (i.e., feelings of being controlled by external forces or self-imposed pressures), competence (i.e., feelings of inefficacy and awkwardness to achieve goals and challenging activities), and relatedness (i.e., feelings of being socially rejected and excluded). Consequently, controlling teaching behaviours would be closely related to both controlled motivation (i.e., behavioural regulation based on experiences of coercion and obligation to think, feel, and behave in a specific way) and amotivation (i.e., the complete lack of self-determination toward the target behaviour), as well as maladaptive affective, behavioural and cognitive outcomes [8,13]. To illustrate, a PE teacher who predominantly uses a coercive language in the lesson, (s)he puts pressure on their students to behave in a teacher-prescribed manner, limiting the number of choices for the tasks (autonomy need satisfaction) and making them feel externally controlled (autonomy need frustration). Similarly, students might have a low sense of efficacy (competence need satisfaction) or develop perceptions of awkwardness of performing the expected activities (competence need frustration), when their PE teacher often demands them to perform an exercise, following rigorous achievement standards for success. Finally, students might not have positive relationships with their peers (relatedness need satisfaction) or they may feel excluded from the group (relatedness need frustration), when their PE teacher ignore their students’ opinions and pressure them to do better than others. Previous studies in the PE context, operationalising controlling teaching behaviour in global terms, have well-documented a negative association of teachers’ controlling teaching behaviours with students’ BPN satisfaction, as well as a positive relationship between this type of teaching behaviours and students’ BPN frustration [2,3,4,5,14,15,16,17,18,19,20,21,22], suggesting the influence of gender on motivational experiences in PE lessons [12,23]. The only existing study that examined internally and externally controlling teaching behaviours from PE teachers showed that although both faces of controlling behaviours related negatively to students’ autonomous and positively to controlled motivation and amotivation, internally controlling were likely to report poor-quality motivation [7].

To the best of our knowledge, there are no validated instruments to date that have examined the external and internal nature of controlling teaching in the PE setting in the SDT framework. So far, a host of measures were developed with each conceptualising PE teachers’ controlling teaching distinctly. Specifically, some slightly modified PE unidimensional scales assessed teachers’ controlling teaching in global terms [24,25], in different social and cultural contexts [20,22,26,27]. Moreover, multidimensional measures of controlling coach behaviours were also developed [28], adapted to PE in the international context [18,29,30], in order to assess the controlling use of rewards and praise, negative conditional regard, intimidation, and, finally, excessive personal control. In addition, other instruments were specifically developed in the PE setting [31,32] to evaluate authoritarian decision-making styles, chaotic communicative strategies, and cold teacher-student interactions as PE teachers’ controlling teaching behaviours. Although such SDT-based measures allowed one to evaluate a large variety of controlling teaching behaviours and made a valuable contribution to the study of controlling teaching in the PE field, they did not take into account the distinction between the external and internal nature of controlling teaching proposed by SDT [10].

In an initial attempt to measure students’ perceptions of external and internal controlling teaching behaviours from PE teachers, De Meyer et al. [7], with the help of 15 experts in SDT, developed the CTS-PE, as a first measure to assess externally controlling teaching and internally controlling teaching (six items per factor). Although this study did not aim to examine its psychometric properties, the scale’s preliminary analyses suggested a nine-item two-factor model that obtained adequate Cronbach’s alpha values for both subscales, after identifying and removing three problematic items. Therefore, there is still a need to gather additional and solid evidence underpinning the instrument’s internal and predictive validity, its measurement invariance across gender, as well as its composite reliability.

### 1.2. The Current Research

Building on SDT, the distinction between externally and internally controlling teaching may provide a deeper understanding of the differential role that teacher’s controlling teaching behaviours could have on students’ motivational experiences, as well as affective, cognitive, and behavioural outcomes in PE [7]. Nonetheless, the absence of well-validated measures to assess both sides of controlling teaching may explain the lack of research on the effects of the external and internal nature of controlling teaching in the PE context. Although De Meyer et al. [7] developed an initial version of the CTS-PE to measure these two dimensions of controlling teaching in PE, this research did not aim to analyse its psychometric properties. Therefore, additional research is required to examine the psychometric properties of the CST-PE. To this end, this research included two different but related studies.

In Study 1, we aimed to examine, via exploratory factor analysis (EFA), the internal composition and structure of the initial pool of 12 items comprising the original version of the CTS-PE [7]. In accordance both with the tenets outlined by SDT [10] and De Meyer et al.’s study [7], we expect to identify a two-factor solution. In Study 2, we aimed to provide validity and reliability evidence for the factor structure previously identified in Study 1 using a different sample of students. For this purpose, a confirmatory factor analysis (CFA) and a multi-group analysis to examine the measurement invariance across gender were, respectively, conducted to provide internal validity evidence. Next, a series of internal consistency analyses were computed to provide reliable evidence of the CTS-PE. Finally, structural equation modelling (SEM) was performed to give predictive validity evidence. In line with SDT assumptions [10] and following previous studies [3,4,5,7,17,20,21], we hypothesised that both externally and internally controlling teaching would positively and significantly predict BPN frustration and would negatively and significantly predict BPN satisfaction.

## 2. Study 1

### 2.1. Materials and Methods

#### 2.1.1. Participants

The sample included 118 male and 123 female secondary school students (*n* = 241), aged from 12 to 16 years old (*M* = 14.04, *SD* = 1.54), from a public secondary school belonging to one medium-sized city in the northeast of Spain. Regarding their educational level, 43 students were in first grade of compulsory secondary education, 81 were in second grade, 24 were in third grade, 38 were in fourth grade, and 55 students were in first grade of post-compulsory secondary education. Most of the students were Caucasian and of middle social, cultural, and economic class. The students received two weekly lessons of compulsory PE with a duration of 60 min. The classes were given by two different PE specialist teachers (2 men) who claimed to have obtained a Bachelor of Science in Physical Education and Sport Sciences and a Professional Master’s program of Education (post-primary PE). This research obtained approval by the Committee for Clinical Research of Aragon (PI15/0283).

#### 2.1.2. Measures

##### Externally and Internally Controlling Teaching in PE

To measure students’ externally and internally controlling behaviours from their PE teachers, a version of the CTS-PE [7] adapted to the Spanish context was used. The instrument is preceded by the stem “My PE teacher…’ and followed by 12 items (six items per factor) that assess externally controlling teaching (e.g., “Yells when I am not doing what (s)he wants me to do”) and internally controlling teaching (e.g., “Pays less attention to me when I disappoint him/her”). Responses to each item were rated on a 5-point Likert-type scale ranging from 1 (not at all true for me) to 5 (very true for me).

#### 2.1.3. Design and Procedure

A cross-sectional design was used to examine the psychometric properties of the CTS-PE [33]. The guidelines described by the International Test Commission [34] were followed to translate the CTS-PE into Spanish. First, the CTS-PE was translated using a back-translation technique. Two groups composed of two translators, respectively, with previous experience in SDT frameworks and the translation of psychometric instruments, completed the translation of the CTS-PE from English to Spanish, and from Spanish to English again. Second, the degree of accuracy of both translations was qualitatively judged by the two groups of translators. Third, and prior to the administration of the instrument to the totality of the students, this was completed by a small group of students (*n* = 10) to ensure the correct understanding of the items.

On the other hand, the researchers contacted the school board and PE teachers at each school involved for their collaboration in this research. Only those students who returned the parents-signed written informed consent participated in this study. Prior to completing a paper-and-pencil questionnaire, the researchers explained to the students that their participation was voluntary and confidential. The questionnaire was administrated in a quiet classroom environment in the absence of the PE teacher and in the presence of the researchers, who were available to answer any question that the participants may have. The average time taken to complete the set of questionnaires was approximately 15 min.

#### 2.1.4. Data Analysis

Prior to data analysis, we detected 12 missing values, which were removed. To evaluate the robustness of running an EFA, the data were inspected by the Kaiser-Meyer-Olkin (KMO) statistic, which is appropriate with values over 0.80 [35], and Bartlett’s sphericity test. Once these criteria were meet, the EFA was performed using the principal component analysis with the promax oblique rotation, setting the kappa parameter at 4 [35]. This type of rotation was used given that it was expected that the underlying dimensions of the controlling teaching would be interrelated. The criterion established by Hair et al. [35], which indicates that an item represents the target factors when its primary loading is higher than 0.50 and its secondary loading up to 0.30, was used to interpret the solution of the resulting items. Additionally, a qualitative analysis of the content for those items with a poor psychometric performance was carried out to complement the quantitative results from EFA. All statistical analyses were performed using SPPS software version 23.00 (IBM Inc., Chicago, IL, USA) [36].

### 2.2. Results of Study 1

The results from both the Kaiser-Meyer-Olkin statistics (KMO = 0.91) and Bartlett’s sphericity test (χ^2^ [*df* = 66] = 1850.30, *p* < 0.001) gathered evidence to support the robustness of running an EFA in the CTS-PE. This last analysis provided a two-factor solution that accounted for 64.77% of the item’s total variance. Table 1 shows that most items had adequate factor loadings (i.e., primary factor loading over 0.50 with secondary factor loading below 0.32) in their respective hypothesised factor. Nonetheless, it is worth noting that item 12 loaded less than 0.50 on both factors, while item 1, item 2, and item 11 obtained a secondary factor loading over 0.30.

After the identification of problematic items, their content was qualitatively analysed by a group of experts in the SDT framework to redraft or remove them from the final version of the instrument. Although item 1 (i.e., “Punishes me”) was initially proposed for the externally controlling teaching factor, its high factor loading in the internally controlling teaching subscale suggests that the concept of punishment could be understood by students in a differentiated way. Most students may perceive that the use of punishments focused not only on shouting and verbal reprimands (i.e., externally controlling teaching), but also on signs of disapproval and withdrawal of attention (i.e., internally controlling teaching). Item 2 (i.e., “Threatens to give bad grades when I do not cooperate”) proposed for the externally controlling teaching factor, loaded also in the internally controlling teaching factor, which might suggest that the threatens to give bad grades by the teacher could be ambiguous for most students. Getting bad grades could be interpreted as an incentive to behave adequately (i.e., externally controlling teaching), but also as a way of appealing to feelings of self-esteem (i.e., internally controlling teaching) given the importance to get good grades for most of the students. Item 11 (i.e., “Act strictly when I disappoint him/her”) proposed for the internally controlling teaching factor, loading also in the externally controlling teaching factor. This might suggest that act strictly could be confusing for most of the students given that it could also be complemented by strategies focused on command, directives, or expressions like “you should” (i.e., externally controlling teaching) in PE lessons. Item 12 (i.e., “shows that [s]he is personally hurt when I do not meet his/her expectations”), initially proposed for the internally controlling teaching factor, did not load in any factor, suggesting that their content could not be understood by students as a controlling teaching behaviour. Taking into consideration the quantitative results and the qualitative interpretation of the items, we decided to remove all problematic items from the final version of CTS-PE. Therefore, an eight-item two-factor solution was proposed for further analyses in Study 2.

### 2.3. Discussion of Study 1

The purpose of Study 1 was to explore the internal composition and structure of the initial pool of 12 items proposed for the CTS-PE [7]. This study extended previous research in the PE field by examining the psychometric properties of this scale.

The results from both the EFA and the qualitative analysis of item content supported an eight-item two-factor solution. Four items belonged to the externally controlling teaching subscale, while the four other items to the internally controlling subscale. This solution was similar to that obtained in the preliminary analyses conducted by De Meyer et al. [7]. Although both studies agreed with the identification of item 1 and item 11 as problematic, this research also identified two problematic items (i.e., item 2 and item 12) that were not found in De Meyer et al.’s study [7]. In fact, De Meyer et al. [7] identified item 7 as problematic, while that item revealed good psychometric properties in this study. These specific differences regarding the psychometric performance of these two items could be due to the distinctive characteristics of the samples of both studies [37]. Anyway, it was unnecessary to rewrite the four items that obtained poor psychometric properties given that, according to Hair et al. [35] and Kline [38], each latent variable under study was represented by at least three items.

## 3. Study 2

### 3.1. Materials and Methods

#### 3.1.1. Participants

A total of 968 secondary school students (479 boys and 489 girls), aged between 12 and 18 years old (*M* = 14.09, *SD* = 1.52), who were enrolled in five different public secondary schools from one medium-sized city situated in the northeast of Spain, participated in this study. With respect to their educational level, 166 students were in first grade of compulsory secondary education, 318 were in second grade, 122 were in third grade, 144 were in fourth grade, and 218 were in first grade of post-compulsory secondary education. Most students were Caucasian and belonged to a middle social, cultural, and economic class. Regardless of their educational level, all students received two one-hour weekly PE sessions, which were taught by nine different PE specialist teachers (eight men and one woman). They self-reported holding a Bachelor of Science in Physical Activity and Sport Sciences and a Professional Master’s program of Education (post-primary PE).

#### 3.1.2. Measures

##### Externally and Internally Controlling Teaching in PE

To assess the students’ perception of externally and internally controlling teaching adopted by PE teachers, we used the resulting eight items (four items per factor) identified in the EFA from Study 1.

##### BPN Satisfaction and Frustration in PE

To assess students’ perceptions of autonomy, competence, and relatedness need satisfaction in PE, we used the Spanish PE version of the Basic Psychological Needs in Exercise Scale [39]; while to assess the students’ perception of autonomy, competence, and relatedness need frustration in PE, we used a Spanish version of the Basic Psychological Need Satisfaction and Frustration (BPNSNF; Chen et al., 2015), adapted to PE context [40]. The two instruments are preceded by the stem “In my PE lessons…” and include four items per factor to measure autonomy satisfaction (e.g., “I feel that the activities I do in PE fit in with my interests”), competence satisfaction (e.g., “I feel that in PE I perform the activities effectively”), relatedness satisfaction (e.g., “I feel that in PE lessons I can communicate openly with my classmates”), autonomy frustration (e.g., “I feel pressured to do too many things”), competence frustration (e.g., “I have serious doubts about whether I can do exercises well”), and relatedness frustration (e.g., “I feel that classmates who are important to me are cold and distant towards me”), respectively. Both instruments are rated on a 5-point Likert-type scale ranging from 1 (strongly disagree) to 5 (strongly agree). In this study, adequate reliability scores were found for autonomy (α = 0.83; ρ = 0.83; ϖ = 0.84, AVE = 0.55), competence (α = 0.82; ρ = 0.83; ϖ = 0.85, AVE = 0.55) and relatedness (α = 0.80; ρ = 0.80; ϖ = 0.82, AVE = 0.51) need satisfaction, as well as for autonomy (α = 0.85; ρ = 0.84; ϖ = 0.87, AVE = 0.58), competence (α = 0.89; ρ = 0.89; ϖ = 0.89, AVE = 0.67), and relatedness (α = 0.90; ρ = 0.90; ϖ = 0.90, AVE = 0.69) need frustration.

#### 3.1.3. Design and Procedure

A cross-sectional design was adopted to provide validity and reliability evidence for the eight-item solution of the CTS-PE [7]. Data collection was completed following a similar procedure as the one described in Study 1, underscoring the voluntary participation of the students in this second study. The time average spent to administrate the questionnaire was approximately 30 min.

#### 3.1.4. Data Analysis

Prior to data analysis, 21 missing values were found and removed. To provide internal validity evidence of the CTS-PE, a CFA and a multi-group analysis were conducted. To perform a CFA, the maximum likelihood estimation method together with 5000-bootstrap samples were used due to the violation of the multivariate normality assumption (Mardia’s coefficient = 29.31, *p* < 0.01) [37]. The goodness of fit was assessed using different fit indexes: chi-squared and degree of freedom (χ^2^/*df*) coefficient, Comparative Fit Index (CFI), Tucker-Lewis Index (TLI), Incremental Fit Index (IFI), Standardised Root Mean Square Residual (SRMR), and Root Mean Square Error of Approximation (RMSEA) with its 90% confidence interval (90%CI). The model fit was evaluated by values below 5 for χ^2^/*df,* above 0.95 for CFI, TLI, and IFI in conjunction with values less than 0.080 and 0.060 for SRMR and RMSEA, which indicate a good fit to data [41]. Standardised residual covariances are suitable with absolute values below 2.58, while standardised regression weights are appropriate with values above 0.50 [35]. Inter-factor correlations display an adequate level of conceptual divergence with values as high as 0.85 [38].

To examine the measurement invariance across gender, a multi-group analysis was conducted in accordance with the methodological approach described by Putnick and Bornstein [42]. This proposal tests the robustness of four progressively constrained models to examine configural invariance (i.e., no equality constraints), metric invariance (i.e., equal item loadings), strict or scalar invariance (i.e., equal item loading and item intercepts concurrently), and strict of error variance invariance (i.e., equal item loading, item intercepts, and item error variance concurrently). The difference between the two multi-group models was assessed based on the differences of CFI and RMSEA value. A value equal to or lower than 0.010 in CFI, together with a value equal to or lower than 0.015 in RMESEA between two progressively constrained models, indicates no differences among them, the tenability of equality constraints and, therefore, the instrument’s invariance assumption (Putnick & Bornstein, 2016). To gather the instrument’s reliability evidence, Cronbach’s alpha (α), McDonald’s omega (ω), Raykov’s composite reliability (ρ) coefficient, and average variance extracted (AVE) were estimated. While Cronbach’s alpha, McDonald’s omega, and Raykov’s coefficient are adequate with values above 0.70 [43], AVE is acceptable with values equal to or greater than 0.50 [35].

In order to provide predictive validity evidence of the CTS-PE, a SEM analysis was used following the two-step approach proposed by Kline [38]. The first step consisted of a CFA to test the robustness of the measurement model with four latent variables of this study. The second step included the examination of the hypothesised model. The relationships between externally and internally controlling teaching and BPN satisfaction and frustration were tested to endorse predictive validity evidence. To this end, the error terms corresponding to BPN satisfaction and frustration were correlated following previous research [17,21], and gender was introduced as a covariate [12,23]. This analysis was performed using the maximum likelihood estimation method in conjunction with the bootstrapping approach with 5000 iterations given the absence of multivariate normality (Mardia’s coefficient = 67.26, *p* < 0.01) [37]. Finally, descriptive statistics and differences by gender for the variables under study were computed. To conduct the data analyses, SPPS software, version 23.00 [36], and IBM SPSS AMOS software, version 23.00 [44] were used.

### 3.2. Results of Study 2

#### 3.2.1. Confirmatory Factor Analysis

**Figure 1:**
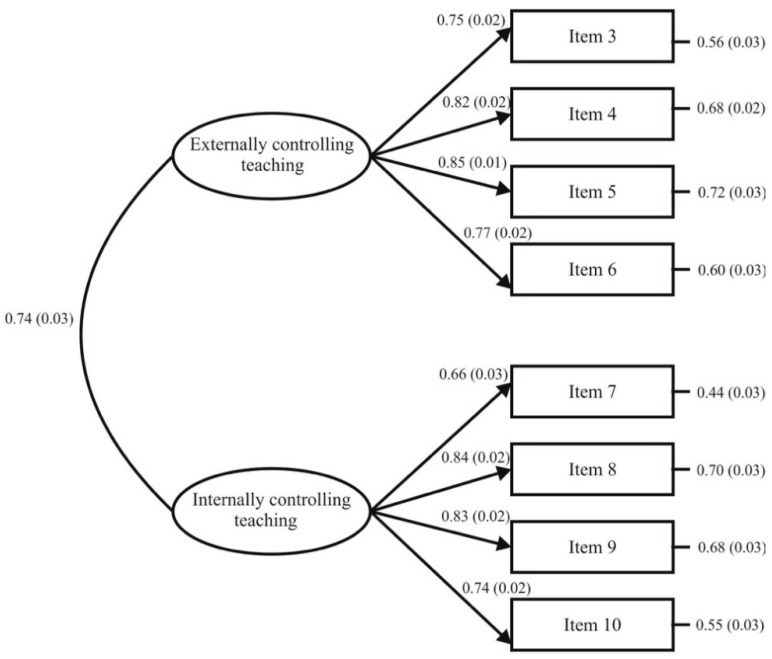
Results from CFA for the CTS-PE. *Note*: The ellipses represent each latent factor and the rectangles symbolize every item. The numbers in parenthesis show the standard error computed by bootstrapping.

The eight-item two-factor correlated model obtained a good fit to the data: χ^2^ (*n* = 968, 19) = 76.29, *p* < 0.001; χ^2^/*df* = 4.02; CFI = 0.99; TLI = 0.98; IFI = 0.99; SRMR = 0.025; RMSEA = 0.056 (90% CI = 0.043–0.069, *p* = 0.215). Standardised residual covariances ranged from −1.51 to 1.58. Figure 1 shows that standardised regression weight values were between 0.66 and 0.85, each being statistically significant (*p* < 0.001). The correlation among externally and internally controlling teaching was 0.74.

#### 3.2.2. Measurement Invariance across Gender

Table 2 shows differences less than 0.010 in CFI values accompanied by differences in RMSEA values lower than 0.015 between each two constrained models. Thus, these results provided evidence of measurement invariance of the CTS-PE across gender.

#### 3.2.3. Reliability Analysis

Acceptable internal consistency values were found for the externally controlling teaching (α = 0.92; ρ = 0.88; ω = 0.87; AVE = 0.64) and the internally controlling teaching (α = 0.85; ρ = 0.85; ω = 0.84; AVE = 0.59) factors.

#### 3.2.4. Structural Equation Modelling Analysis

The first step of SEM analysis was to test the measurement model with four latent variables, which yielded a good fit to the data: χ^2^ (*n* = 968, 81) = 366.35, *p* < 0.001; χ^2^/*df* = 4.32; CFI = 0.96; TLI = 0.95; IFI = 0.96; SRMR = 0.038; RMSEA = 0.060 (90% CI = 0.054–0.067, *p* = 0.003). Standardised regression weights were between 0.66 and 0.85 (*M* = 0.78), each reaching the level of statistical significance (*p* < 0.001). Correlations among factors ranged from −0.67 to 0.74 (see Table 3). These results provided evidence supporting the robustness of the measurement model tested.

**Figure 2:**
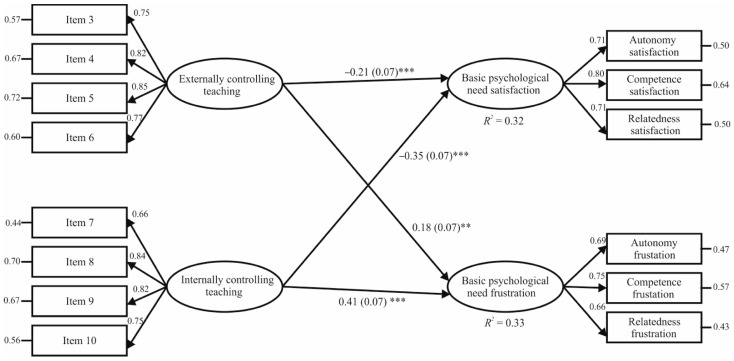
Relationships between externally and internally controlling teaching and need satisfaction and frustration in PE. *** *p* < 0.001, ** *p* < 0.01. *Note:* The numbers in parenthesis display the standard error estimated by bootstrapping. The correlation between externally and internally controlling teaching was *r* = 0.74, while the correlation of error terms between BPN satisfaction and frustration was *r* = −0.58.

The second step of SEM was to analyse the proposed theoretical hypothesis model. This obtained appropriate goodness-of-fit measures: χ^2^ (*n* = 968, 81) = 366.35, *p* < 0.001; χ^2^/*df* = 4.52; CFI = 0.96; TLI = 0.95; IFI = 0.96; SRMR = 0.038; RMSEA = 0.060 (90% CI = 0.055–0.067, *p* = 0.003). Figure 2 displays that, after controlling for gender, both externally and internally controlling teaching positively predicted BPN frustration (*β* = 0.18, *p* = 0.002; *β* = 0.41, *p* < 0.001), and negatively BPN satisfaction (*β* = −0.21, *p* < 0.001; *β* = −0.35, *p* < 0.001). The total variance explained by this model was 33% and 32% for BPN frustration and BPN satisfaction, respectively.

#### 3.2.5. Descriptive Statistics and Gender Differences among Study Variables

Table 3 shows that mean scores for the target variables were, except for BPN satisfaction, below the midpoint of the measurement scale. Moreover, independent *t*-tests found that while boys scored significantly higher in BPN satisfaction, girls obtained significantly higher values in BPN frustration. Instead, there were no significant differences between boys and girls in the externally and internally controlling teaching values.

### 3.3. Discussion of Study 2

The objective of Study 2 was to examine the psychometric properties of the eight-item two-factor correlated model previously found for the CTS-PE in Study 1. The results offered initial evidence to consider the CTS-PE as the first valid and reliable measure to evaluate students’ perceptions of externally and internally controlling teaching behaviours from PE teachers.

The results from the CFA for the CTS-PE provided psychometric support for the eight-item two-factor correlated model. The standardised residual covariances did not exceed 2.58 as an absolute value, implying the absence of misspecification in the instrument’s internal structure, as well as of substantial discrepancies between the theoretical two-factor correlated model tested and the data observed. Furthermore, all standardised regression weights displayed values over 0.50 with each reaching the statistical significance level, indicating thus that each item adequately captured the meaning of the subscale aiming to measure. The inter-factor correlation showed a moderate association between the externally and internally controlling teaching subscales, consistent with the one in De Meyer et al.’s study [7]. This moderate correlation value supports not only the appropriate degree of theoretical discrimination among both factors, but also the SDT’s assumption of the existence of two distinguishable but also related faces of controlling teaching in the PE context [7,10].

The findings that emerged from the gender invariance analysis, displayed that the eight-item two-factor correlated model was invariant between groups of male and female secondary school students. This means that the CTS-PE held equally for boys and girls, allowing one to examine the possible gender differences in students’ perceptions of externally and internally controlling behaviours from teachers in the PE setting. It is important also to highlight that these results are, particularly, interesting because they offered the first evidence to date of the measurement invariance across gender of this instrument. With regard to the instrument’s reliability analysis, satisfactory values were found for each of the two subscales. The Cronbach’s alpha values obtained were similar to those reported by prior research [7], while the estimation of McDonald’s omega, Raykov’s coefficient, and AVE gathered further reliability evidence for the CTS-PE and extended previous findings.

Consistent with the SDT’s tenets and prior research conducted in PE [3,4,5,7,17,20,21], the results from SEM provided predictive validity evidence for the CTS-PE. Controlling teaching behaviours, regardless of its external or internal nature, were positively associated with BPN frustration and negatively with BPN satisfaction. It should be emphasised that teachers’ internally controlling teaching behaviours had a greater predictive effect than externally controlling teaching behaviour on students’ perceptions of BPN satisfaction and frustration in their PE lessons. These findings suggest that teaching behaviours focused primarily on guilt-induction, through facial and verbal expressions of disappointment and withdrawal of attention, may cause students to feel more self-controlled in learning (i.e., autonomy need frustration), more ineffective to perform the proposed activities (i.e., competence need frustration), and more rejected by their teacher (i.e., relatedness need frustration) than the exposure to externally controlling teaching strategies. Similarly, these findings reflected that the exposure by students to controlling teaching behaviours and, particularly, internally controlling teaching behaviours, contributed to undermining their perception of autonomy, competence, and relatedness need satisfaction when they participated in PE. These results are in line with the only existing study that also found that internally controlling behaviours from PE teachers were particularly associated with poor-quality motivation [5]. As a whole, these results suggested that although both controlling teaching practices have been associated with maladaptive outcomes on students’ psychological experiences in PE lessons, PE teachers should, particularly, refrain from using internally controlling strategies when teaching students.

## 4. General Discussion

The objective of this SDT-based research was to examine the psychometric properties of the CTS-PE [7] using a sample of Spanish secondary school students. The results from these two sequential studies provided evidence to consider the CTS-PE, in its eight-item two-factor correlated model, as a valid and reliable measure to assess students’ perceptions of externally and internally controlling teaching behaviours from PE teachers. To the best of our knowledge, there were no psychometrically robust instruments to date to assess the external and internal nature of teachers’ controlling teaching behaviours in the PE context, which would explain the little that we do know about the impact of externally and internally controlling teaching on the students’ motivational processes involved in PE. Consistent with the only existing study [7], our findings highlight that both controlling practices and, particularly, internally controlling teaching behaviours are related to maladaptive motivational outcomes. Therefore, PE teachers should avoid the use of controlling strategies when teaching students.

This research offered some interesting and useful findings, but also had some limitations that should be acknowledged. Firstly, as a non-probabilistic sampling method was applied in this study to recruit the study sample, the results obtained should be interpreted carefully, which makes it impossible to generalise them to a broader population. Additional research is required to examine the psychometric properties of the CTS-PE in other education stages (e.g., primary school), students enrolled in different types of school (e.g., private), and students from distinct social, cultural, and economic contexts to extend the psychometric body of evidence for this scale. Secondly, this research only examines the psychometric properties of the CTS-PE in the Spanish context, hence the need to develop future studies that provide new validity and reliability evidence of this instrument in other linguistic contexts (e.g., English, French, Estonian or Portuguese). Thirdly, although the hypothesized model, to test the predictive validity evidence, was based on the SDT framework, this research adopted a cross-sectional design, not allowing us to establish causal relationships between the variables studied. Further research in the PE context could use longitudinal or experimental designs to explore the impact of externally and internally controlling teaching behaviours on students’ need satisfaction and frustration, motivation, and (mal)adaptive affective, behavioural, and cognitive outcomes.

### Implications for Practice

The CTS-PE will enable us to more deeply and comprehensively examine the differentiated role that the external and internal faces of controlling behaviours from PE teachers could have on motivational processes (BPN satisfaction and frustration and behavioural regulations) and affective, behavioural, and cognitive outcomes exhibited by students in their PE lessons. Furthermore, this will allow us to provide PE teachers with helpful and valuable information on motivational risks associated with externally and internally controlling behaviours in order to refrain from using these motivating practices, which may suppose an advance in the improvement of the quality both of the instructional practices in the school PE, and the initial and continuous education programmes for PE teachers. Thus, the issue of teacher’s externally and internally controlling behaviours deserves a particular attention in planning both initial education programmes for PE pre-service teachers and continuous professional development programmes for in-service teachers.

## 5. Conclusions

The present research provided psychometric evidence in support of the CTS-PE, in its eight-item two-factor correlated model, as the first valid and reliable measure to assess Spanish students’ perceptions of externally and internally controlling teaching behaviours from teachers in the context of the secondary school PE. Therefore, this scale can contribute to a better understanding of potential risks associated with the development of externally and, particularly, internally controlling behaviours from teachers on students’ psychological and motivational outcomes in PE lessons. Further studies are required to determine whether the two distinguishable faces of controlling teaching behaviours influence the students’ motivational processes and dynamics involved in the PE lessons in the same way.

## Figures and Tables

**Table 1:** Results from the EFA of the CTS-PE.

My Physical Education Teacher… [Mi Profesor/a de Educación Física…]	ECT	ICT
1. Punishes me [Me castiga]	0.84	−0.36
2. Threatens to give bad grades when I do not cooperate [Me amenaza con una mala nota cuando no me implico en los ejercicios/actividades]	0.83	−0.33
3. Counts down aloud to make sure that I persist [Cuenta hacia atrás en voz alta para asegurarse de que lo intento una y otra vez en los ejercicios/actividades]	0.75	0.27
4. Threatens with sanctions when I am not doing what (s)he tells me to do [Me amenaza con castigarme cuando no estoy haciendo lo que él/ella me dice que haga]	0.78	0.20
5. Threatens that we will not do any fun activities when I do not cooperate [Amenaza con no hacer ninguna actividad divertida cuando no me implico en los ejercicios/actividades]	0.79	0.21
6. Yells when I am not doing what (s)he wants me to do [Me grita cuando no estoy haciendo lo que él/ella quiere que haga]	0.75	0.20
7. Is less friendly with me when I do not do the things his/her way [Es poco amistoso/a conmigo si no hago las cosas a su manera]	0.01	0.67
8. Pays less attention to me when I disappoint him/her [Me presta menos atención cuando le decepciono]	0.33	0.63
9. Makes me feel guilty when I disappoint him/her [Me hace sentir culpable cuando le decepciono]	0.17	0.67
10. Often shows that (s)he is disappointed in me [A menudo muestra que está decepcionado conmigo]	0.26	0.75
11. Acts strictly when I disappoint him/her [Es muy estricto/a cuando le decepciono]	0.44	0.69
12. Shows that (s)he is personally hurt when I do not meet his/her expectations [Se muestra dolido/a cuando no cumplo sus expectativas]	0.01	0.45

*Note*: ECT = Externally controlling teaching; ICT = Internally controlling teaching. Items of the Spanish version are found in square brackets.

**Table 2:** Measurement invariance across gender.

	χ^2^	*df*	χ^2^/*df*	CFI	TLI	IFI	SRMR	RMSEA (90%CI)	MC	Δχ^2^	Δ*df*	ΔCFI	ΔRMSEA
1. Configural invariance	85.04	38	2.24	0.989	0.983	0.989	0.027	0.036 (0.026–0.046)	-	-	-	-	-
2. Metric invariance	96.33	44	2.19	0.988	0.984	0.988	0.028	0.035 (0.026–0.045)	2 vs. 1	11.29	6	−0.001	−0.001
3. Strict invariance	103.81	52	2.00	0.988	0.987	0.988	0.028	0.032 (0.023–0.041)	3 vs. 2	7.48	8	0.000	−0.003
4. Strong invariance	141.00	60	2.35	0.981	0.982	0.981	0.033	0.037 (0.029–0.045)	4 vs. 3	37.19 ***	8	−0.007	0.005

*Note*: MC = Models comparison, vs. = versus. *** *p* < 0.001.

**Table 3:** Descriptive statistics, gender differences, and correlations among study variables.

	Total Sample				Boys	Girls	*t*-Tests		Correlations			
	Range	*M* (*SD*)	γ_1_	γ_2_	*M* (*SD*)	*M* (*SD*)	*t*	*d*	1.	2.	3.	4.
1. Externally controlling teaching	1–5	1.95 (1.08)	1.10	0.29	1.94 (1.05)	1.96 (1.10)	−0.14	0.01				
2. Internally controlling teaching	1–5	1.44 (0.72)	0.78	−0.02	1.41 (0.69)	1.46 (0.74)	−0.90	0.06	0.74 ***			
3. Need satisfaction	1–5	3.59 (0.80)	−0.69	0.82	3.73 (0.76)	3.45 (0.82)	5.43 ***	0.36	−0.47 ***	−0.51 ***		
4. Need frustration	1–5	1.98 (0.83)	1.25	1.81	1.88 (0.77)	2.08 (0.88)	−3.71 ***	0.24	0.49 ***	0.55 ***	−0.67 ***	

*** *p* < 0.001.

## Data Availability

Data availability on request due to restrictions privacy or ethical.
